# It’s Late, But Everything Comes Next: Creative Aging in the Modern United States

**DOI:** 10.1093/geroni/igaa057.2965

**Published:** 2020-12-16

**Authors:** Shayna Gleason

**Affiliations:** University of Massachusetts, Boston, Boston, Massachusetts, United States

## Abstract

The emergence of “creative aging” programs, or fine arts programs exclusively for older adults, invites analysis of these new institutions and their influence on the social elements of the aging process. While past studies have demonstrated the cognitive and health-related benefits of arts participation in old age, little research has examined how participation might influence the older person’s self-esteem or perceptions of aging. The present study draws on ethnographic methods including participant observation at eight creative aging programs, six semi-structured in-depth interviews with teaching artists leading these programs, and content analysis of paintings and vignettes made by participants. The results show the observed creative aging programs to have a unique, cyclical pattern of discourse characterized alternately by older adults’ recurring self-deprecation and the affirming responses of instructors. This pattern of interaction renders such programs sites for the contestation of negative popular discourses around aging.

